# Epithelioma of the Cervix, Complicating Labor

**Published:** 1885-11

**Authors:** Carlton C. Frederick

**Affiliations:** Assistant in Obstetrics, Niagara Medical College


### * Epithelioma of the Cervix, Complicating Labor.

By Carlton C. Frederick, M. D.

Assistant in Obstetrics, Niagara Medical College.

Epithelioma of the cervix, complicating labor, is a rare occurrence in the experience of the general practitioner. Therefore, it may be of interest to the readers of this journal, as well as of practical use, for me to give the history of a case, and briefly recount the experiences and teachings of eminent obstetricians in dealing with this grave complication of labor.

Mrs. C., æt. 29, previously having borne two children, was taken in labor on Saturday evening, the 15th of September. I was not called till early the following Monday morning. Up to this hour, her pains had been mild at long intervals, such as are usual in the first stage. I had never seen the woman before. The pains, at the time of my first visit, were quite severe about every five minutes. On examination, the os was found dilated to about the size of a silver dollar, thick, hard, ragged and nodular to the touch, the membranes ruptured, and the head presenting in the first position. On entering the room, I had noticed a very offensive odor, and on withdrawing my fingers, found them covered with blood, fetid muco-pus, and shreds of broken-down tissue.

* Read before the Buffalo Medical and Surgical Association.

I ascertained that she was somewhere between the seventh and eighth month of pregnancy; also, that, previous to conception and ever since, she had had a bloody and, at other times, a purulent fetid discharge, which had been worse during the past few months. A second examination, more carefully made, convinced me that on both sides of the cervix there was some comparatively healthy tissue, it not feeling so hard or nodular, the greater part of the growth being confined to the anterior and posterior portions. Between pains, I put her in Sim's position and obtained a fair view of the os, especially the anterior and lateral portions. It looked raw and bleeding, with many gray shreds of dead tissue adhering. In its appearance, I could think it nothing but a malignant growth.

She told me that her children had both been small. In view of this fact, together with the more healthy condition of the cervix laterally, I determined to wait for dilatation and assist it as much as possible by mechanical means. I watched it for two hours and could see some progress. The pains became much stronger and forced the head, with the thick cervix drawn over it, well down into the superior straits. As she was in much constant distress, I gave her morphine hypodermically, ¼ grain, and repeated it in one hour. This gave her a rest for some time, during which I introduced a Barnes dilator. I then put a colperynteur into the vagina, and inflated it for the purpose of retaining the Barnes dilator in position in the cervix. Before this procedure, I had washed out the vagina with a 1-2000 solution of corrosive sublimate. I kept her under the influence of morphine well into the night. Every 3 or 4 hours I removed the dilators and found that progress was being made. Finally, the largest Barnes dilator was too small, and I introduced a small-sized colperynteur. After the cervix had been dilated to about 3 inches in diameter, I gave her no more morphine, and, in a couple of hours, pains began to come harder and more frequently. After two hours of this, with only a small gain, I had about despaired of it, and thought to get help and proceed by some means to deliver. Three ways presented: either to nick the diseased tissues at several points and thus allow the head to come out, or to put on forceps and force a way out, or to make version and deliver. At this time, several hard pains succeeded each other at short intervals, and during the last and hardest the tissues gave way, on the left side principally, and the head entered the pelvis. A small child was soon born, apparently lifeless, but was soon resuscitated.

After delivery of the placenta, the uterine cavity and vagina were irrigated with a 1-2000 solution of corrosive sublimate, hot, for the purpose of checking considerable bleeding, which presumably came from the rent in the cervix. As the bleeding was too much, in one-half hour I again irrigated the vagina with hot water, and the oozing was controlled.

The sublimated vaginal douche was ordered given every 12 hours. On the second day, the discharge became very offensive, and the temperature rose to 102½°. The douche was given every six hours, and the temperature dropped to 100°, above which it did not again rise. On the third day, several large shreds came away, and I removed several more with the finger—evidently some of the cancerous tissue broken down by pressure. She has made a good convalescence so far. The child had sore eyes for several days. I have not yet examined the cervix, but will be glad to report to you the further history of the case later.

In the short time I have since had to look over the literature of this subject, I have found considerable matter of practical and statistical value. Much of it that comes from the German obstetricians, I have borrowed from an article on the same subject by Dr. R. W. Stewart, of Cincinnati, in the *American Journal of Obstetrics* for June, 1885.

Lusk says: "The existence of pregnancy hastens the development of cancer, the more rapid growth of which is probably referable to the increased vascularity of the uterus and to the correspondingly augmented activity of its nutritive processes. If the morbid process has involved the entire portio vaginalis, or even extended into the os internum, the inelastic tissue of the cancerous growth has replaced the expansile muscular fibres, and an opening of sufficient calibre for the passage of the fœtus can only be produced by rupture and contusion of the degenerated and unyielding cervix. The consequence of the excessive pressure to which the cervix is subjected during labor is necrosis of the contused tissues, which is frequently followed by fatal septicæmia. The prognosis is doubtful for both mother and child. The latter is imperiled by its liability to premature expulsion, and by the mechanical obstruction to its birth. The mother's life is not only shortened by the rapidity of the cancerous growth usually induced by pregnancy, but is jeopardized by her increased liability to abortion, post-partum hemorrhage and puerperal fever."—(*Science and Art of Midwifery*, p. 510.)

This is a plain statement of the dangers attending these cases. How are they to be treated best, when, and with what results?

Dr. Lusk says about treatment: "In cases where the disease is confined to the cervical portion, either amputation or excision should be performed, preferably at about the fourth month. Abortion does not necessarily follow. In advanced cases, where the carcinomatous process has invaded the contiguous tissues, operative interference should be postponed until the end of gestation. An extensive removal of diseased tissues during pregnancy exposes the mother to the immediate danger of premature labor and subsequent septicæmia, while it is hardly possible to do the work so effectively as to procure a free outlet for the child. Upon the advent of labor, if the child be living, the Cæsarian section certainly holds out the hope of saving one life, and probably does not greatly increase the peril to which the other is exposed."—(*Science and Art of Midwifery*, p. 510.)

Playfair's opinion is that, "If it is possible, the disease should be removed, as much as can be safely done, during pregnancy, which should be brought to an end before the full period. During labor, incisions should form a preliminary to any subsequent proceedings that may be necessary, as they are not liable, at the worst, to increase the risks the patient has to run, and they may possibly avert more serious operations. These failing, forceps, Cæsarian section or craniotomy may be resorted to.—(*Midwifery*, 3d American Ed., pp. 349 and 350.)

Schroeder, after examining the possible and probable dangers to mother and child, gives his ideas of treatment as follows—(*Lehrbuch d. Geburtshülfe*, p. 496):

"The removal of the degenerated mass is chiefly to be recommended, which removal can be made much more easily and with less danger during pregnancy. Consequently, it is best to operate, when possible, a short time before the expected beginning of labor. If labor has begun, however, so much of the new growth is to be removed by the fingers or the sharp curette, or, if necessary, by cutting instruments, as will permit of the application of forceps or the performance of version. If one proceed energetically in this manner and remove the new growth, the Cæsarian section, which is extremely dangerous to the mother, may be avoided. The Cæsarian section is to be practiced when the child is still alive and sufficient space cannot be procured for the application of forceps or the performance of version. In such cases, the life of the child is of greater importance than that of the mother, because the latter is otherwise inevitably lost."

Spiegelberg thinks that "The difficulties which carcinoma uteri produces in labor, depend altogether upon the longitudinal and peripheric extent of the disease. When the degeneration is limited to the lips, and has left the angles free, so that in the cervical wall some dilatable portions still remain, the difficulties may wholly fail, dilatation and expulsion proceeding undisturbed.

"When the whole os, as well as the vaginal portion, is involved, the upper and larger part of the cervix becomes dilated, but the ring produces an obstinate resistance. It becomes, under the pressure of the child, somewhat wider, but still insufficiently so; is broken through, destroyed, or a metritis is developed, which, as a rule, diminishes the expulsive power. As a rule, the expulsion occurs rapidly and the consequent necrosis hastens the lethal termination, unless the absorption of the products of decomposition brings on puerperal septicæmia. He recommends dilatations by the rubber bags or the making of incisions into the infiltrated portions. When these are insufficient, the child must be extracted with forceps if living, or by craniotomy if dead.

"The incisions are liable to a further tearing into the tissues—how far, it is impossible to foretell—so that the expulsion or extraction of the head must be guarded by the hand in order to avoid rupture of the uterus.—(*Lehrbuch der Geburtshülfe*, 1878.)

Scanzoni says: "It is a most dangerous complication of labor. When the infiltration is not too great, the birth may occur naturally from dilatation of the healthy portion. Women with cervical carcinoma, as a rule, are attacked by a virulent form of puerperal fever and generally die. That the prognosis for the child is very bad."

Herman, after assenting to the principles of treatment before enumerated, says, that, in his experience, delivery by forceps, when dilatation has progressed far enough, is preferable to version.—(*Trans. of the Obstet. Soc. of London*, vol. xx., p. 233.)

Leishman, Cohnstein, Meadows, Cazeaux, Ramsbotham, Bedford and other obstetric writers say what practically amounts to the same thing. The older writers, however, say less upon the subject than the later, and give greater preference to the Cæsarian section.

Now, a great deal of the uncertainty manifested by them has given place to more confident and scientific methods of procedure, as is attested by the following cases reported in brief:

I. Frommel reports a case of Schroeder's in the Berlin clinic, where, the child being dead, the operator broke away large pieces of the growth with his hands, and delivered by version.

The patient was discharged in ten days, and subsequently died. —(*Ztsch. f. Geburtsh. und Gynæk.*, Bd. v., p. 158.)

II. Dr. Fordyce Barker had three cases of spontaneous delivery, in all of which the mother survived the puerperal period. These are very exceptional, however.

III. Spiegelberg. Woman, æt. 37; second child; anterior lip showed kidney-shaped tumor, 5 cm. thick by 8 cm. long, hard and unyielding; removed a large portion of the tumor by the galvano-cautery, followed by incisions into the os; forceps applied; mother died on the seventh day; child died after a few days from trismus.

IV. Dr. Herman—(*Obstet. Trans.*, London, vol. xxiii., p. 308):

Woman, 37; fifth child; anterior lip involved. The diseased tissue was freely cut away with scissors and the actual cautery, and delivery effected by forceps. The cancer had invaded the anterior vaginal wall, and a vesico-vaginal fistula formed here. Phlebitis set in, and patient died on the eighteenth day.

V. Dr. Herman—(same source):

Diseased tissues removed by the ecraseur, the fingers and scissors; hemorrhage trifling; delivery by forceps. The mother recovered.

VI. Dr. Edis—(*Am. Jour. Obstet.*, vol. xvi., p. 186):

Cancer involving nearly the whole circumference of the cervix and a greater part of the posterior vaginal wall; os dilated to the size of a five-shilling piece; Cæsarian section made; child asphyxiated, but recovered. The mother recovered, and when seen six months after, the disease had made but little progress.

VII. Dr. J. M. Arnott—(*Med. and Chirurg. Trans.*, vol. xxxi., p. 37):

Woman, 38 years old; multipara, anterior lip enlarged, hard, and presenting a growth the size of a large walnut, projecting toward the right. Tumor removed by scissors, followed by normal birth; both mother and child alive. After several months, a similar growth developed from the posterior lip, followed by death of the woman.

VIII. Michaelis—(*Neue Zeitschr. f. Geburtsh.*, Bd. iv., p. 176):

Woman, 30; fifth child; cauliflower growth from anterior lip, filling the whole vagina. Tumor removed by scissors; child delivered by version, found dead. A similar growth afterwards appeared on the posterior lip, followed by death.

IX. Dr. Mundé—(*Am. Jour. Obstet.*, vol. xv., p. 917):

Multipara; hemorrhage profuse; cervix taken away by the galvano-cautery. Upon sixth day after operation, abortion occurred. At second month, patient recovered.

X. Dr. Mundé—(*Am. Journal Obstet.*, vol. xv., p. 917):

Woman, 41 years; disease of posterior lip. Not knowing she was pregnant, the growth was removed by the galvano-cautery. Later history showed that she must have been in the fourth month at the time of the operation. At the end of the eighth month, there had been no return of the disease, and she was still pregnant.

XI. Godson—(*Obstet. Trans.*, London, vol. xxv.):

Patient not suspected to be pregnant. Removed the whole cervix. About ten days after, put a catheter into the uterus in demonstrating a point to an assistant, followed by abortion.

XII. Dr. Spaeth—(*Wochenbl. der Zeitschr. Wiener Aerzte*, 1855, No. 14):

Cancer of posterior lip, removed at second month; delivered at term; mother and child lived.

XIII. Savory—(*Obstet. Trans.*, London, vol. xvii., p. 82):

Whole cervix diseased; tumor removed two and one-half months before term; labor at term; good delivery; child alive. This patient became pregnant again in two years, the cancer having made rapid growth. Delivered by version; child dead. Mother died in thirteen days.

XVI. Galabin—(*Obstet. Trans.*, London, vol. xviii., p. 237):

Epithelioma of whole cervix; pregnancy of one month; removed whole growth. At term, os was dilated by rubber dilators, and a living child delivered by version. Considerable hemorrhage after delivery, but the woman is known to have lived three and one-half months after delivery, at least.

XV. Dr. Stewart (*Am. Jour. Obst.*, vol. xviii., p. 577):

Woman, æt. 39; fourth child; epithelioma of anterior lip, projecting down into the vagina; removed the tumor with the ecraseur and fingers; slight hemorrhage; unable to apply forceps; delivered by version; child apparently asphyxiated, but resuscitated. Seven weeks after labor, Dr. S. reports child doing well, but the disease returning at the original site — the anterior lip.

Herman collected ten cases, in which the disease was removed during pregnancy. In one case, it was at the end of the first month. The patient went to term, but the disease returned before delivery.

One case, operated in second month, went to term; easy labor; four cases operated on about fifth month; one aborted the day following; one went to about seven and one-half months; two went to term. All recovered well.

One case operated on at sixth month, Douglas' cul-de-sac being opened; aborted eleven days after; child dead.

One case operated on at seventh month; labor one week later.

One case operated on at eighth month; dead child born eight days after.

One operated near the end of pregnancy; labor followed in five days.—(*Obstet. Trans.*, vol. xx., p. 223.)

Puchelt collected 27 cases, in which 5 mothers died during labor, 9 shortly after labor, 10 recovered, and the history of the remaining 3 is unknown.—(Quoted by Scanzoni, *Lehrbuch d. Geburtsh.*)

Scanzoni had 7 cases: 4 died during labor from hemorrhage; 3 a few days later from puerperal fever. All the children were born dead.—(*Lehrb. d. Geburtsh.*, Bd. ii., p. 237.)

Cohnstein collected 134 cases, and finds that, "Of 127 cases, the anterior lip was involved 12 times; the posterior lip,

7 times; the os externum, 22 times; the side of the cervix, once; the whole cervix, 85 times.

"In 100 births, 68 occurred at term; premature birth or abortion occurred in 30; 2 cases of delayed labor: one at ten and one-half, the other at seventeen months.

"Premature labor happened 8 times at seventh month; 5 times in eighth month; once in the ninth month, and once in nine and one-quarter months.

"Abortions occurred 3 times in third month; 4 times in fourth month; once at four and one-half months; once at fifth month; 3 times at sixth month; twice, unknown.

"Of 126 mothers, 54, or 42.9 per cent., recovered from labor; 72, or 57.1 per cent., died in labor or soon after.

"Of the 72, 54 died at term, 38 after delivery, and 16 undelivered.

"The exact time of death of 51 is known: 31 during, or immediately after, labor; 2 each on the first, second, fourth, fifth and twenty-first days; 5 times on the third day; once each on the sixth, seventh, eighth, fourteenth and eighteenth days.

"Of those mothers who lived, it is known that 13 lived from four weeks to six months; and 1 each, eight and ten and one-half months; and 1 each, two and four years.

"Of 110 children born, 42, or 36.2 per cent., were born alive; 74, or 63.8 per cent., were born dead; of these 74, 17 died from craniotomy or embryotomy; 1 at sixth month after Cæsarian section; 1 from produced abortion; 14 wherein the mothers died undelivered (13 at term, and 1 at seventh month); 6 were born alive, but died soon after birth."

To recapitulate, it would appear, in the light of the best authorities, that the plan of treatment may be thus tabulated:

1. When the disease is not too extensive, it should be removed early in pregnancy.

2. When the disease is extensive, it should be removed near or at the time of labor.

3. "When this cannot be done, the safety of the mother is best consulted by bringing the pregnancy to an end as soon as possible."—(*Herman.*)

4. When labor has come on, expansion of the os should be aided by numerous small incisions in its circumference, and the use of rubber dilators.

5. Dilatation of the os being in progress, and uterine action is not sufficient to force the head through the cervical canal, forceps or version may be resorted to.

6. When dilatation cannot take place after removal of the diseased tissues, the incision of the os and use of dilators, either from the size of the tumor or rigidity of the tissues, Cæsarian section should be done.

